# Mesenteric Meckel's Diverticulum as a Missed Diagnosis in a Child With Recurrent Rectal Bleeding: A Report of a Challenging Case

**DOI:** 10.7759/cureus.113755

**Published:** 2026-07-31

**Authors:** Bhavanam Abhishek, Vedaant Parekh, Subrat K Mohanty, Varsha M Totadri, Harish Chandra Tudu, Pradeep K Jena, Swati Das

**Affiliations:** 1 General Surgery, Kalinga Institute of Medical Sciences, Bhubaneswar, IND; 2 Paediatric Surgery, Kalinga Institute of Medical Sciences, Bhubaneswar, IND; 3 Paediatric Surgery, Vardhman Mahavir Medical College & Safdarjung Hospital, New Delhi, IND; 4 Radiodiagnosis, Kalinga Institute of Medical Sciences, Bhubaneswar, IND

**Keywords:** ectopic gastric mucosa, meckel’s diverticulum, mesenteric, obscure gastrointestinal bleeding, paediatric

## Abstract

Meckel's diverticulum is a common congenital anomaly of the gastrointestinal tract and is a remnant of the omphalomesenteric duct*.* One of the classical identification and diagnostic criteria of Meckel's diverticulum is its location along the anti-mesenteric border of the ileum. Mesenteric Meckel's diverticulum (MMD) is an unexpected occurrence, rarely reported, with confounding diagnostic tests due to the atypical location, often forming a gray zone in diagnosis due to its similarity to an enteric duplication cyst. A 1.5-year-old male child presented to the pediatric surgery outpatient department (OPD) with complaints of intermittent episodes of painless rectal bleeding and melena for five months. On examination, the child was pale, and the abdomen was soft, with no tenderness or palpable mass. Digital rectal examination showed dark melena stool staining. The patient had a history of prior admissions in outside hospitals, where he underwent an upper gastrointestinal (UGI) endoscopy and colonoscopy, both of which were normal. A Meckel's scan done in an outside hospital was positive, and therefore, the patient underwent a diagnostic laparoscopy in the center, wherein no Meckel's diverticulum nor other bowel abnormalities were identified. Thereafter, the patient was referred to our hospital due to persistence of symptoms. Evaluation was repeated to ensure a thorough investigative protocol. Repeat UGI endoscopy and colonoscopy revealed no abnormality. A red blood cell (RBC) tagged radionuclide scan (Tc99) was done, which showed pooling of blood in the small bowel (possibly ileum) and hepatic flexure. The child underwent an exploratory laparotomy, which showed an obscure diverticulum in the mid-ileum, on the mesenteric side, buried within the mesentery. The diverticulum had a wide base with luminal communication with adjacent ileum, with a separate vascular supply within the mesentery, suggestive of an MMD. The patient underwent resection of the Meckel's diverticulum with end-to-end anastomosis of the ileum. The postoperative period was uneventful, and the child thereafter had no further episodes of rectal bleeding or melena. Histopathology confirmed a 3.5 cm Meckel's diverticulum with ectopic gastric mucosa. Anti-mesenteric location of a diverticulum is a classical diagnosis of Meckel's diverticulum. The rare entity of mesenteric location can confound the diagnosis due to similarity with an enteric duplication cyst. While clinical presentation remains the same irrespective of mesenteric or anti-mesenteric location, it can often be missed intraoperatively due to the unexpected location. Diagnostic evaluation and planned surgical intervention are prudent to ensure complete resection and clinical recovery.

## Introduction

During fetal life, the yolk sac communicates with the developing gut via the omphalomesenteric duct, which normally obliterates between the fifth and eighth week of gestation [1]. Failure of obliteration results in Meckel’s diverticulum (MD), the most common congenital gastrointestinal anomaly, occurring in approximately 0.6%-4% of the population [2,3]. MD is a true diverticulum containing all layers of the intestinal wall and is typically located on the anti-mesenteric border of the ileum, 60-100 cm proximal to the ileocecal valve [1,4].

Clinical presentation ranges from asymptomatic incidental findings to complications such as hemorrhage, intestinal obstruction, perforation, and intussusception [3,5]. The classic “rule of twos” (2% prevalence, 2 feet from the ileocecal junction, 2 inches long, and symptomatic by age 2 years) remains a useful heuristic despite recognized exceptions [2,6].

Mesenteric MD (MMD), arising from the mesenteric rather than anti-mesenteric border of the ileum, is an exceptionally rare variant. Since its first description in 1941, only 32 cases have been reported, including 14 pediatric cases [7]. Its atypical location poses a diagnostic challenge, as it may be missed during laparoscopic inspection focused on the anti-mesenteric border. MMD may also mimic an enteric duplication cyst because of its mesenteric position and variable vascular supply [8,9].

We report a 1.5-year-old boy with recurrent painless rectal bleeding in whom MMD was missed on initial diagnostic laparoscopy despite a positive Meckel’s scan, ultimately requiring exploratory laparotomy for definitive diagnosis and treatment. This case underscores the need for a high index of suspicion for mesenteric variants and careful inspection of both mesenteric and anti-mesenteric borders during surgical evaluation of obscure gastrointestinal bleeding.

This submitted case report was presented as an oral paper presentation at IAPSCON (Indian Association of Pediatric Surgeons' National Conference) 2025 in Puri, Odisha, in November 2025.

## Case presentation

**Figure 1:**
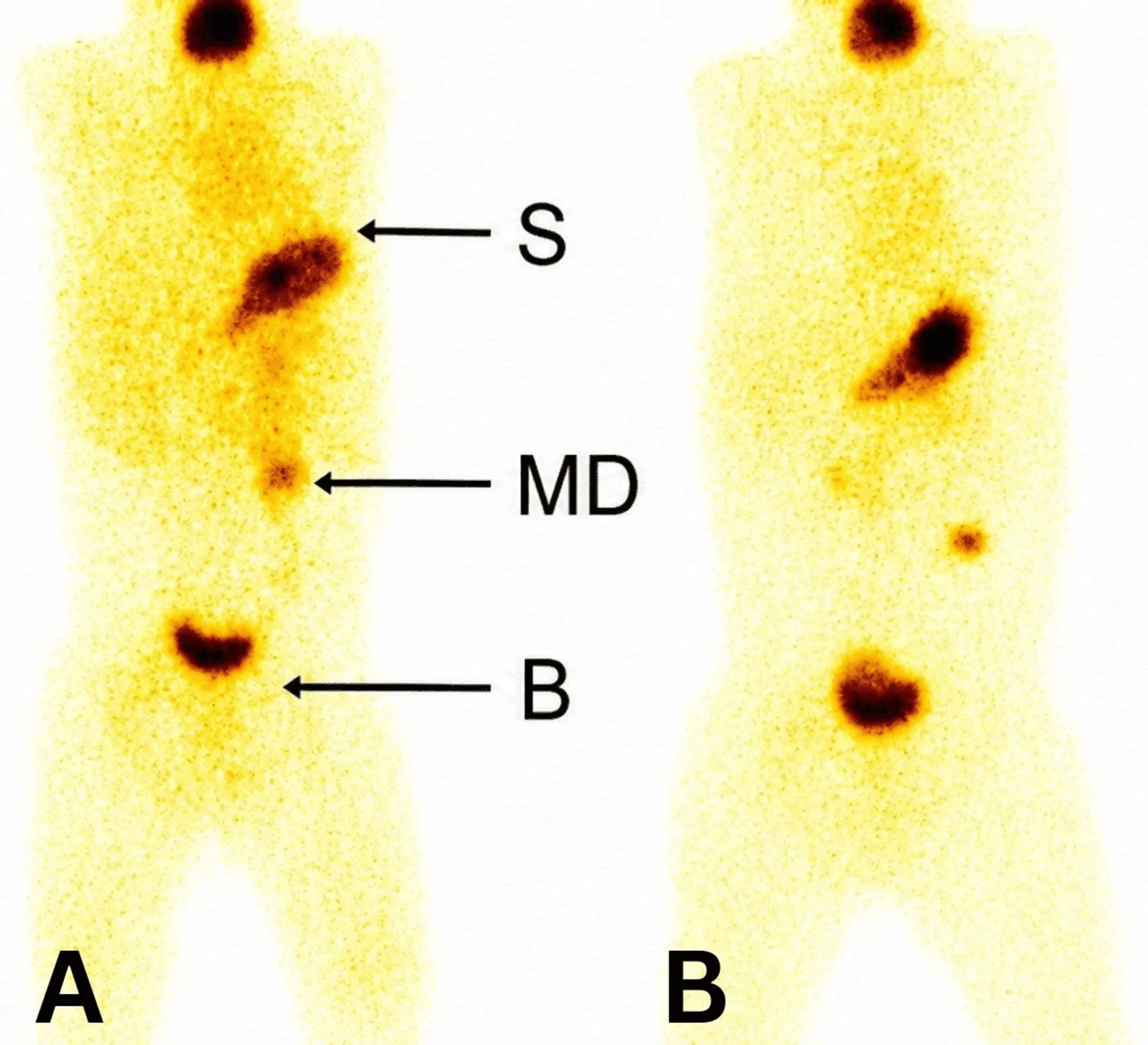
Initial technetium-99m pertechnetate scintigraphy (Meckel's scan) from the outside facility. (A) Static anterior abdominal image demonstrating normal physiological radiotracer uptake in the stomach (S) and urinary bladder (B), with a persistent abnormal focal area of radiotracer accumulation in the mid-abdomen corresponding to a Meckel's diverticulum (MD) containing ectopic gastric mucosa. (B) Corresponding delayed static anterior abdominal image demonstrating persistent radiotracer uptake in the same mid-abdominal focus, confirming ectopic gastric mucosa within the Meckel's diverticulum, with physiological radiotracer uptake again seen in the stomach and urinary bladder.

A 1.5-year-old male child presented to the pediatric surgery outpatient department with a five-month history of intermittent painless rectal bleeding (hematochezia) and occasional passage of black tarry stools (melena). There was no history of abdominal pain, vomiting, fever, weight loss, or any other significant medical illness. The child had previously been evaluated at an outside pediatric facility, where a technetium-99m pertechnetate (Meckel’s) scan was reported as positive (Figure 1: image photographed from the report, as outside images could not be procured from the system directly from a different facility).

Based on this finding, he underwent diagnostic laparoscopy; however, no MD or other bowel abnormality was identified, and the source of bleeding remained elusive. The postoperative recovery was uneventful, and the child remained asymptomatic for approximately three months following discharge.

Three months later, he developed recurrent episodes of painless rectal bleeding and melena and was evaluated at a second pediatric center. Repeat investigations, including upper gastrointestinal endoscopy, colonoscopy, and contrast-enhanced computed tomography (CECT) of the abdomen and pelvis, failed to identify the source of bleeding. As the symptoms improved spontaneously, he was discharged on supportive treatment with vitamin supplementation. However, one month later, the child experienced another episode of bleeding and was subsequently referred to our institution for further evaluation and management.

On presentation to our institution, the child was hemodynamically stable but had clinical pallor. Abdominal examination revealed a soft, non-tender abdomen with no palpable masses or organomegaly. Digital rectal examination demonstrated blood-stained stool with evidence of melena. Laboratory investigations revealed microcytic hypochromic anemia, with hemoglobin levels of 7 g/dL (normal: 10.5 to 15 g/dL as per laboratory parameters), for which the child received a packed red blood cell (RBC) transfusion.

**Figure 2:**
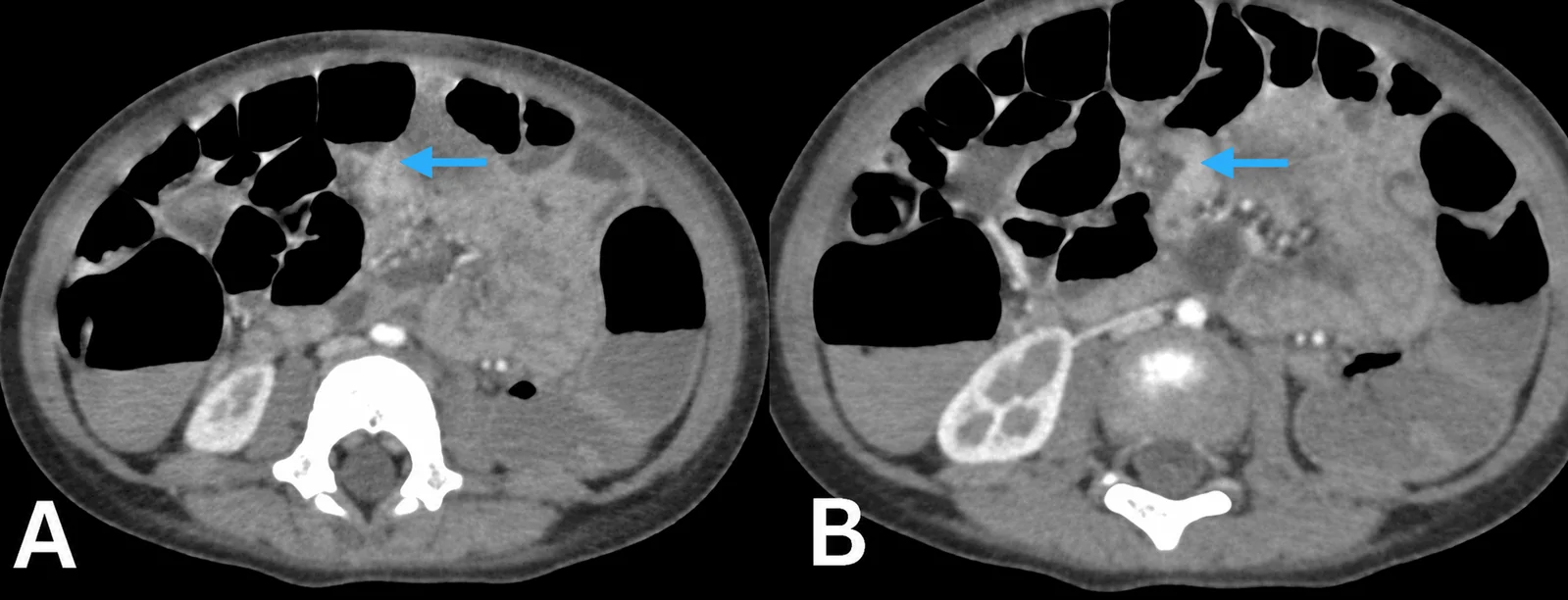
Contrast-enhanced computed tomography (CT) enterography demonstrating a blind-ending tubular structure arising from the mid-ileum (blue arrow in A and B). Axial image in (A) showing a suspicious tubular structure, further well-defined in (B), showing a well-demarcated tubular diverticular outpouching (blue arrows) arising from the small bowel.

Given the recurrent nature of the bleeding and the inconclusive findings of previous evaluations, a comprehensive reassessment was undertaken. After careful review of the prior investigations and operative records, a technetium-99m RBC scintigraphy was performed. The scan demonstrated tracer accumulation in the ascending colon and hepatic flexure; however, the findings remained non-specific and failed to accurately localize the source of bleeding. In view of the persistent clinical suspicion and the equivocal radionuclide findings, a CECT scan of the abdomen and pelvis with CT enterography was subsequently obtained. This revealed a 1.8 cm blind-ending tubular structure arising from the mid-ileum, suggestive of a diverticulum and highly suspicious for MD (Figure 2).

**Figure 3:**
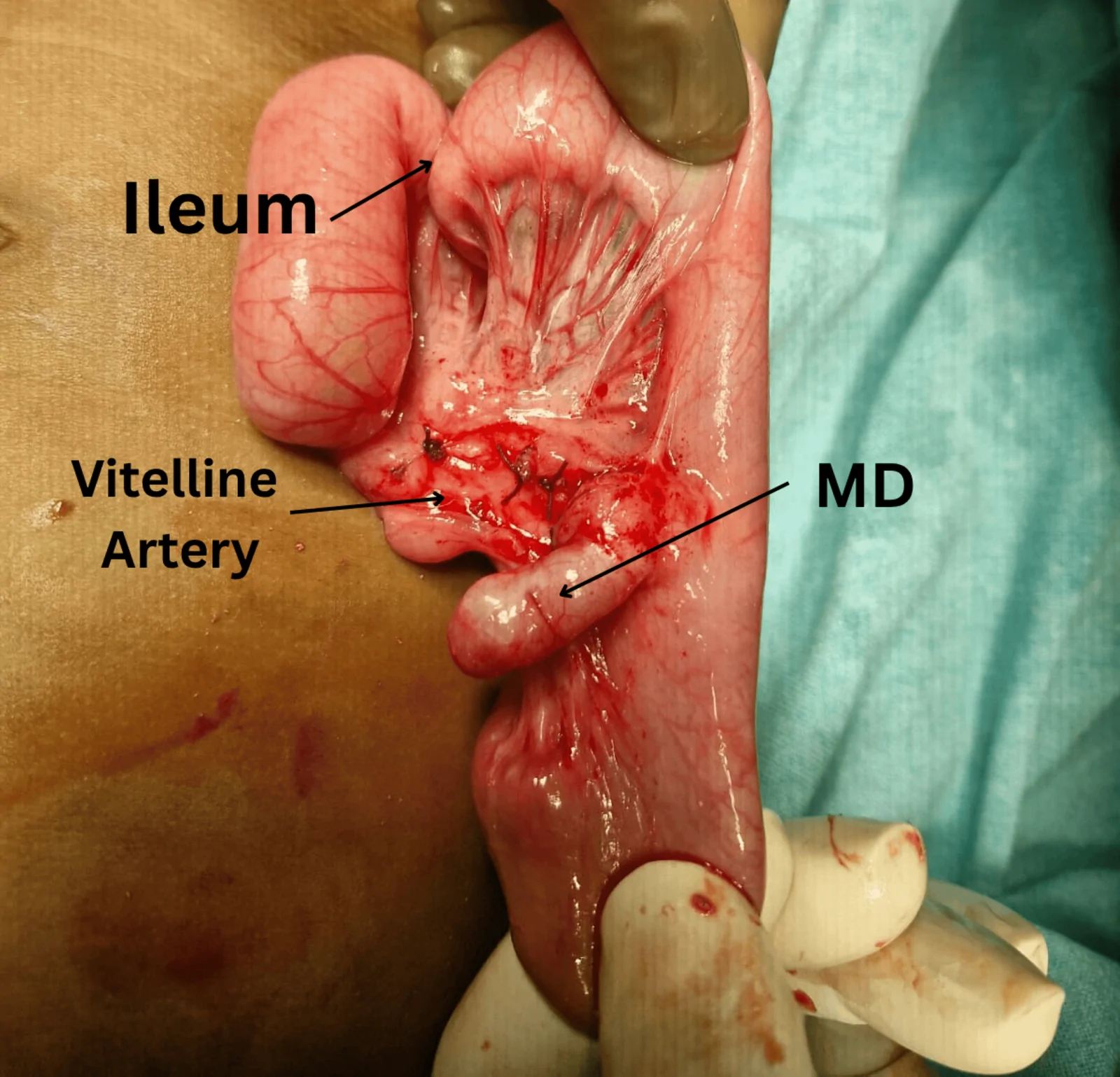
Intraoperative view of the mid-ileum, demonstrating a 3.5 cm mesenteric Meckel’s diverticulum (MMD) arising directly from the mesenteric border, complete with its own distinct vitelline artery.

Considering the recurrent episodes of gastrointestinal bleeding, multiple prior hospital admissions, positive functional imaging, and radiological evidence of a small bowel diverticulum, exploratory laparotomy was undertaken. Intraoperatively, meticulous examination of the entire ileum was performed, including careful inspection of both the anti-mesenteric and mesenteric borders. A diverticulum measuring approximately 3.5 cm in length was identified on the mesenteric border of the ileum, approximately 20 cm proximal to the ileocecal junction (Figure 3).

The diverticulum was completely buried within the mesentery, making identification difficult on routine inspection and likely accounting for its failure to be detected during the previous diagnostic laparoscopy. It possessed a broad base with direct communication to the ileal lumen and was supplied by a distinct vascular pedicle arising from the mesenteric vessels, consistent with a persistent vitelline artery (Figure 3). These findings confirmed the diagnosis of an MMD. No evidence of inflammation, perforation, bowel obstruction, intussusception, or other associated pathology was identified.

Given the broad-based attachment and mesenteric vascular supply, a segmental ileal resection incorporating the diverticulum was performed, after skeletonizing and doubly ligating the feeding vessel, followed by primary end-to-end ileo-ileal anastomosis. The postoperative course was uneventful. Enteral feeds were initiated on postoperative day 2, and the child tolerated them well. He was discharged on postoperative day 6 in stable condition.

**Figure 4:**
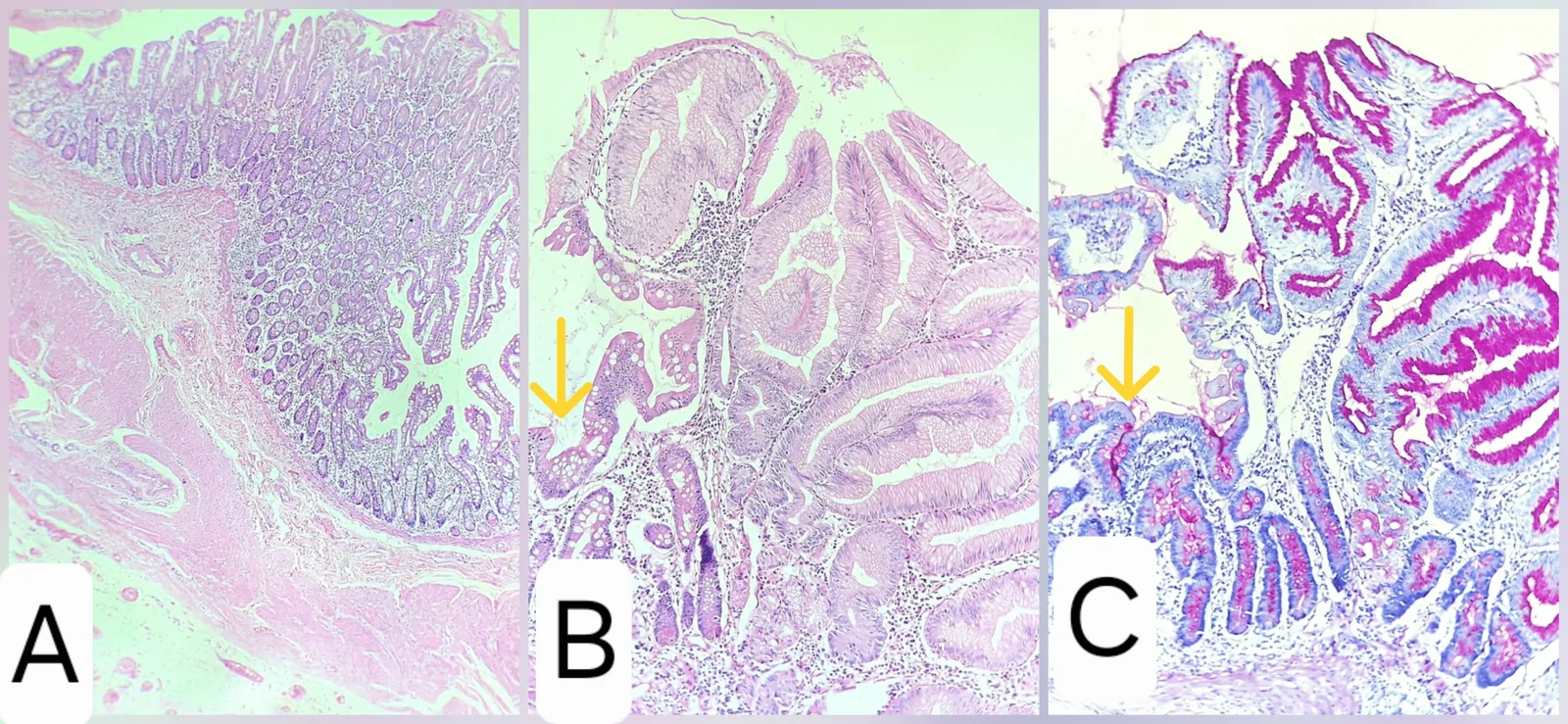
(A) Section from the MMD showing all the layers (H&E; 40X); (B) section from the diverticula showing both small intestinal mucosa and gastric mucosa (yellow arrow) (H&E; 400X). (C) Magenta-colored neutral mucin in lining epithelium in the apex of the mucosal tissue confirming its gastric origin (PAS; 400X). MMD: mesenteric Meckel’s diverticulum; PAS: Periodic acid-Schiff

Histopathological examination confirmed the diagnosis of MMD. Microscopic sections from the diverticulum revealed a true diverticulum, which was identified by the presence of all the layers of intestine. The lining epithelium was mostly small intestinal mucosal tissue with interspersed gastric mucosal tissue (Figures 4A, 4B). Special stain using Periodic acid-Schiff (PAS) highlighted the neutral mucin in the lining epithelium, confirming its gastric nature (Figure 4C). No evidence of metaplasia, dysplasia, or malignancy was seen.

At the one-year follow-up, the child remained completely asymptomatic, with no further episodes of hematochezia or melena. Growth and developmental milestones were age-appropriate, and no postoperative complications were observed (Table 1).

**Table 1:** Timeline of the clinical course. GI: gastrointestinal; CT: computed tomography; RBC: red blood cell

Time point	Event
Month 0	Initial symptoms begin: intermittent painless rectal bleeding (hematochezia) and melena.
Month 1	Outside hospital evaluation: technetium-99m pertechnetate (Meckel’s) scan reported as positive.
Month 1.5	Diagnostic laparoscopy performed at outside facility-no Meckel’s diverticulum identified. Postoperative recovery uneventful.
Months 1.5-4.5	Asymptomatic period (approximately 3 months).
Month 4.5	Symptoms recur: painless rectal bleeding and melena.
Month 5	Second outside hospital evaluation: repeat upper GI endoscopy, colonoscopy, and contrast-enhanced CT (CECT) of the abdomen and pelvis-all normal. Symptoms improve spontaneously; discharged with vitamins.
Month 6	Referral to our hospital. RBC tagged radionuclide scan (Tc-99m) shows pooling of blood in small bowel (possibly ileum) and hepatic flexure. CECT enterography reveals a 1.8 cm blind-ending tubular structure arising from the mid-ileum.
Month 6	Exploratory laparotomy performed. Mesenteric Meckel’s diverticulum (MMD) identified on the mesenteric border of the mid-ileum, buried within the mesentery. Resection of diverticulum with ileum and end-to-end anastomosis done.
Postoperative day 2	Resumption of oral intake.
Postoperative day 6	Discharged home.
One-year follow-up	Asymptomatic, no further bleeding, normal growth and developmental milestones.

## Discussion

MD results from incomplete obliteration of the omphalomesenteric (vitelline) duct between the fifth and seventh weeks of embryogenesis [1,2]. The classical description-a true diverticulum located on the anti-mesenteric border of the distal ileum-is so consistent that many surgeons do not routinely inspect the mesenteric border when searching for an MD [3,4]. MMD challenges this assumption. With only 32 reported cases since 1941 (14 pediatric) (Table 2), MMD represents a rare but clinically significant anatomical variant that can elude even experienced surgeons [5-7].

**Table 2:** Summary of previously reported pediatric mesenteric Meckel’s diverticulum (MMD) cases. ICV: ileocecal valve; RLQ: right lower quadrant; RIF: right iliac fossa

Author (year)	Age/sex	Presentation	Procedure	Distance from ICV	Ectopic mucosa	Outcome
Chaffin (1941) [10]	8 y/M	Melena, emesis, pain	Small bowel resection	--	Gastric	Recovered
Kurzbart et al. (2002) [11]	3 m/--	Patent omphalomesenteric duct	Diverticulectomy	40 cm	Not reported	Recovered
Sarioglu-Buke et al. (2008) [12]	7 m/M	Painless rectal bleeding	Segmental resection	40 cm	Gastric	Recovered
Manukyan et al. (2009) [13]	15 y/F	Abdominal pain, perforation	Segmental ileal resection	90 cm	Gastric + pancreatic	Recovered
Wani et al. (2013) [14]	9 y/M	RLQ pain (appendicitis mimic)	Appendectomy + diverticulectomy	90 cm	Absent	Recovered
Singh et al. (2013) [15], Case 1	2 y/F	Umbilical discharge	Small bowel resection	40 cm	Absent	Recovered
Singh et al. (2013) [15], Case 2	2 y/M	Episodic bleeding	Small bowel resection	30 cm	Gastric	Recovered
Singh et al. (2013) [15], Case 3	3 y/M	Umbilical pain	Small bowel resection	30 cm	Gastric	Recovered
Mohanty et al. (2014) [16]	16 m/F	Rectal bleeding	Small bowel resection + anastomosis	60 cm	Gastric (antral)	Recovered
Abdul Kader et al. (2019) [17]	10 y/M	Abdominal pain, altered stool	Ileal resection + anastomosis	4 cm	Focal gastric	Recovered
Yagnik and Dawka (2019) [18]	15 y/F	Periumbilical pain	Segmental resection	60 cm	Gastric	Recovered
Yagnik (2021) [19]	14 y/F	Vomiting, RIF pain	Segmental resection	60 cm	Gastric	Recovered
Koetter et al. (2021) [20]	17 m/F	Rectal bleeding	Segmental resection + anastomosis	30-40 cm	Gastric	Recovered
Alkhamisy et al. (2021) [7]	2 y 7 m/M	Painless bleeding	Segmental resection + anastomosis	30 cm	Gastric	Recovered
Present case (2025)	1.5 y/M	Painless bleeding, melena	Wedge resection + primary repair	20 cm	Gastric (oxyntic)	Recovered, no recurrence

Several hypotheses have been proposed to explain the aberrant mesenteric position. The most widely accepted theory involves persistence of a short vitelline artery, which creates a mesodiverticular band tethering the diverticulum to the mesentery. As the small bowel elongates during development, this band pulls the diverticulum toward the mesenteric border rather than allowing it to remain on the anti-mesenteric side [8,9-13]. Alternative explanations include congenital or inflammatory adhesions between the vitelline duct and the ileal mesentery, or asymmetric involution of the omphalomesenteric duct [9,10,12]. In our case, the presence of a separate vascular pedicle from the mesenteric vessels strongly supports the first hypothesis.

The clinical presentation of MMD is indistinguishable from that of classical MD. In symptomatic patients, hemorrhage (due to ectopic gastric mucosa) is the most common presentation in children, as seen in our patient [3,5,13-16]. However, the atypical location creates two major diagnostic pitfalls. First, imaging studies such as Meckel's scan may be falsely negative or equivocal. While Tc-99m pertechnetate has a sensitivity of 81%-90% and specificity > 95% for detecting ectopic gastric mucosa within an MD, its accuracy depends on the presence of sufficient parietal cell mass and adequate tracer uptake [6,17,18]. In MMD, the diverticulum may be buried within the mesentery, potentially reducing tracer accumulation due to attenuation or altered blood flow. Moreover, a positive Meckel's scan does not localize the diverticulum to the anti-mesenteric border, and false positives can occur with enteric duplication cysts, vascular malformations, or inflammatory bowel disease [19-21]. In our case, the initial positive Meckel's scan was followed by a negative diagnostic laparoscopy, likely because the surgeon inspected only the anti-mesenteric border.

Second, intraoperative identification remains the gold standard, but it requires a deliberate search of both borders. Our case exemplifies this: the diagnostic laparoscopy at the outside facility was negative, presumably because the diverticulum was completely buried within the mesentery and not visible from a standard laparoscopic approach. This underscores a critical point: a negative anti-mesenteric inspection does not exclude MD [4,6,22].

The mesenteric location of MMD naturally raises suspicion for an enteric duplication cyst, another congenital anomaly that can present with bleeding, obstruction, or a palpable mass [23,24]. Several features help distinguish the two entities (Table 3). MMD is a true diverticulum with a luminal communication to the native bowel (though the communication can be narrow), contains all five intestinal layers, and typically has a separate blood supply from the vitelline artery [1,4,9-12]. Enteric duplication cysts usually share a common muscular wall and blood supply with the adjacent bowel, rarely communicate with the lumen, and may have a separate mucosal lining (often gastric or respiratory) [23,24]. Histopathology in our case confirmed a diverticular structure with all five layers and a clear luminal communication, ruling out a duplication cyst.

**Table 3:** Distinguishing features between mesenteric Meckel’s diverticulum and enteric duplication cyst.

Feature	Mesenteric Meckel's diverticulum	Enteric duplication cyst
Location [4,20]	Mesenteric border of ileum	Mesenteric side, often within leaves of mesentery
Luminal communication [4]	Present (can be narrow)	Usually absent
Wall layers [1,23]	All 5 layers of small intestine	Usually shares muscularis propria with native bowel
Blood supply [1,4]	Separate vitelline artery	Shared with native bowel
Ectopic mucosa [2,5]	Gastric (most common)	Gastric, respiratory, or pancreatic
Presentation [5]	Bleeding, obstruction, perforation	Obstruction, mass, bleeding (less common)

Given the limitations of Meckel's scan and diagnostic laparoscopy, cross-sectional imaging can provide valuable adjunctive information. CECT with CT enterography can sometimes demonstrate a blind-ending tubular structure arising from the small bowel, as seen in our patient [25]. In our case, the CECT revealed a 1.8 cm blind-ending tubular structure arising from the mid-ileum, which prompted the decision to proceed with exploratory laparotomy rather than repeat laparoscopy. Magnetic resonance enterography offers similar sensitivity without ionizing radiation but is less available in emergency settings [15-17,21]. Technetium-99m RBC scintigraphy (as used in our patient) can localize active bleeding but does not identify the underlying structural lesion [26].

Complete surgical resection is the standard of care for symptomatic MD [3,5-8,21-24]. For MMD, a segmental ileal resection with primary anastomosis is generally recommended over simple diverticulectomy, because the diverticulum is often wide-based and intimately associated with the mesenteric blood supply [20,27]. In our patient, we performed a segmental ileal resection with end-to-end anastomosis. Postoperative outcomes are excellent when the diagnosis is made and complete resection is achieved; our patient remained symptom-free at one year.

This case offers several practical lessons. First, MD should remain in the differential diagnosis of obscure gastrointestinal bleeding in children even after a negative diagnostic laparoscopy, especially if a Meckel's scan was positive. Second, surgeons performing laparoscopy for suspected MD must deliberately inspect the mesenteric border of the entire ileum, which may require mobilizing the mesentery or converting to an open approach if the diverticulum is not readily visible. Third, repeat imaging with CECT enterography can help localize a suspected lesion before reoperation. Finally, multidisciplinary communication among pediatricians, radiologists, and surgeons is essential to avoid diagnostic delays.

Limitations

As a single-case report, our findings may not be generalizable. The rarity of MMD precludes large prospective studies, and the true incidence may be higher than reported because many cases likely remain undiagnosed or unreported. Long-term follow-up beyond one year is always advisable.

## Conclusions

MMD is a rare but clinically significant variant that should be considered in children presenting with obscure gastrointestinal bleeding, particularly when a positive Meckel’s scan is not confirmed by anti-mesenteric inspection alone. The diagnosis is often missed during initial laparoscopy because the diverticulum is buried within the mesentery. A negative inspection of the anti-mesenteric border does not exclude MD. Thorough evaluation of both the anti-mesenteric and mesenteric borders during surgical exploration-or use of preoperative CT enterography-is essential to avoid missing this surgically correctable entity. Complete resection leads to excellent clinical outcomes.
